# *Gfap* Mutation and Astrocyte Dysfunction Lead to a Neurodegenerative Profile with Impaired Synaptic Plasticity and Cognitive Deficits in a Rat Model of Alexander Disease

**DOI:** 10.1523/ENEURO.0504-24.2025

**Published:** 2025-03-19

**Authors:** Robert F. Berman, Matthew R. Matson, Angelica M. Bachman, Ni-Hsuan Lin, Sierra Coyne, Alyssa Frelka, Robert A. Pearce, Albee Messing, Tracy L. Hagemann

**Affiliations:** ^1^UC Davis M.I.N.D. Institute, University of California Davis, Davis, California 95816; ^2^Department of Neurological Surgery, University of California Davis, Sacramento, California 95816; ^3^Institute of Molecular Medicine, College of Life Sciences, National Tsing Hua University, Hsinchu 30013, Taiwan; ^4^Waisman Center, University of Wisconsin-Madison, Madison, Wisconsin 53705; ^5^Department of Anesthesiology, University of Wisconsin-Madison, Madison, Wisconsin 53705; ^6^Department of Comparative Biosciences, School of Veterinary Medicine, University of Wisconsin-Madison, Madison, Wisconsin 53706

**Keywords:** astrocyte, hippocampus, innate immune response, long-term potentiation, mitochondria, synapse

## Abstract

Alexander disease (AxD) is a rare neurological disorder caused by dominant gain-of-function mutations in the gene for glial fibrillary acidic protein. Expression of mutant protein results in astrocyte dysfunction that ultimately leads to developmental delay, failure to thrive, and intellectual and motor impairment. The disease is typically fatal, and at present there are no preventative or effective treatments. To gain a better understanding of the link between astrocyte dysfunction and behavioral deficits in AxD, we have recently developed a rat model that recapitulates many of the clinical features of the disease, including failure to thrive, motor impairment, and white matter deficits. In the present study, we show that both male and female AxD model rats exhibit a neurodegenerative profile with a progressive neuroinflammatory response combined with reduced expression of synaptic and mitochondrial proteins. Consistent with these results, AxD rats show reduced hippocampal long-term potentiation and are cognitively impaired, as demonstrated by poor performance in the Barnes maze and novel object recognition tests. The AxD rat provides a novel model in which to investigate the impact of astrocyte pathology on central nervous system function and provides an essential platform for further development of effective treatments for AxD and potentially other neurodegenerative diseases with astrocyte pathology.

## Significance Statement

Alexander disease (AxD) is a fatal neurodegenerative disorder caused by gain-of-function glial fibrillary acidic protein mutations. We have recently developed a *Gfap*^+/R237H^ rat model that demonstrates hallmark astrocyte pathology, myelin deficits, and motor impairment. Here, we show that *Gfap*^+/R237H^ rats exhibit reduced synaptic plasticity and cognitive deficits as additional clinically relevant phenotypes, further demonstrating its utility as a model. Hippocampal transcriptomic analysis in young adult animals reveals a neurodegenerative signature with an innate immune response and loss of synaptic and metabolic gene expression, features that are typically associated with chronic diseases of aging. These results reveal mechanisms by which astrocyte dysfunction leads to learning and memory deficits in AxD and perhaps contributes to other diseases such as Alzheimer's and Parkinson's.

## Introduction

Alexander disease (AxD) is a progressive and generally fatal disorder of the central nervous system (CNS), with a range of clinical phenotypes including cognitive and motor impairments (Messing et al., 2012; Messing, 2019; Hagemann, 2022). In early-onset cases, AxD is associated with prominent white matter deficits, especially in the frontal lobes (van der Knaap et al., 2001), leading to its original classification as a leukodystrophy, whereas later-onset cases are associated with lesions of the posterior fossa (Prust et al., 2011). In all cases, the hallmark pathological feature is the presence of Rosenthal fibers, protein aggregates found within the cytoplasm of astrocytes, particularly in perivascular, subpial, and subependymal locations (Alexander, 1949). Over 90% of cases of AxD result from heterozygous missense mutations in the gene encoding the astrocyte intermediate filament, glial fibrillary acidic protein (GFAP; Brenner et al., 2001). Since this discovery, AxD has become a model system in which to explore the consequences of primary astrocyte dysfunction in the CNS (Messing et al., 2012). Over the past few decades, the role of astrocytes in regulating synapse formation and activity in both development and disease has become an active area of research (Araque et al., 1999; Allen and Eroglu, 2017; Blanco-Suarez et al., 2017). Although deficits in the hippocampal pyramidal layer (CA1) and striatum have been demonstrated in some cases of AxD (Borrett and Becker, 1985), neuronal loss has not been widely reported (Sosunov et al., 2018).

Despite the initial description of AxD as an intellectual disability syndrome (Alexander, 1949), disturbances in cognitive function have received little attention. Generalizations about the prevalence of the cognitive phenotype in different AxD subtypes are frequently stated in print, but it is not clear how often and the degree to which patients are cognitively impaired. Indeed, many questions remain unanswered, including which domains of cognitive function are affected, whether impairments localize to known sites of pathology, and whether cognitive deficits, once characterized, might be useful as outcome measures in the testing of experimental treatments.

We have recently developed a rat model of AxD that demonstrates myelin loss, clinically relevant motor phenotypes, and increased mortality, more closely recapitulating the human disease compared with earlier mouse models (Hagemann et al., 2006; 2021). Previous behavior testing in AxD model mice demonstrated deficits in learning and memory (Hagemann et al., 2013), but these deficits were subtle and strain specific. In this report, we take advantage of the rat model to investigate whether astrocytes compromised by GFAP mutation impair synaptic, neuronal, and cognitive functions.

## Materials and Methods

### Rat model of AxD

The *Gfap*^+/R237H^ rat model of AxD, hereafter referred to as R237H, was generated as previously described (Hagemann et al., 2021) and maintained as heterozygotes on a Sprague Dawley genetic background (Charles River CD IGS rat). All animals were bred at the University of Wisconsin-Madison. For molecular, histological, and physiological analyses, animals were housed under specific pathogen-free conditions in the AAALAC-accredited Waisman Center Rodent Models Core. Rats were cohoused as wild-type (WT) and R237H pairs whenever possible under a 12 h light cycle and fed *ad libitum*. For behavioral phenotyping, rats were bred at the University of Wisconsin-Madison (UW-Madison), and pregnant dams were sent to the University of California Davis (UC Davis) M.I.N.D. Institute Intellectual and Developmental Disabilities Research Center. Animals housed at UC Davis were kept under the same conditions as those in the Waisman Center at UW-Madison (cohoused as WT and R237H pairs under a 12 h light cycle and fed *ad libitum*). All animal studies were approved by the Animal Care and Use Committees at UC Davis or under the College of Letters and Sciences and Vice Chancellor Office for Research at UW-Madison.

### RNA isolation and hippocampal transcriptomics

Male and female littermates of each genotype were killed at 3 or 8 weeks of age by CO_2_ asphyxiation (*N* = 4 per group, 32 samples total), and the hippocampus was collected immediately on ice and frozen (−80°C) before subsequent processing. The hippocampus included Ammon's horn, the dentate gyrus (DG), and the subiculum. The alveus was used as a dorsal boundary to separate the hippocampus from the subcortical white matter, and parahippocampal regions were removed from the ventral/lateral boundaries. Excess white matter from the fimbria and fornix were also removed. RNA was extracted with TRIzol reagent per the manufacturer's protocol (Invitrogen, Thermo Fisher Scientific) and treated with TURBO DNase (TURBO DNA-free Kit, Ambion, Thermo Fisher Scientific), and RNA integrity was determined with an Agilent 4200 TapeStation system for quality control. Libraries were prepared from 1 µg total RNA with integrity numbers (RIN) between 8.4 and 9.3 for sequencing with TruSeq Stranded mRNA Sample Preparation kit (Illumina). Libraries were quantified with PicoGreen reagent (Thermo Fisher Scientific) and assayed with an Agilent TapeStation system to confirm integrity before sequencing with an Illumina NovaSeq X Plus (2 × 150 bp, 70 M reads per sample, two lanes). Base calling was performed using Bcl2fastq (v2.20.0.422), read trimming with Skewer (Jiang et al., 2014), read alignment with STAR (Dobin et al., 2013), expression estimation with RSEM (Li and Dewey, 2011), and differential expression estimation with EdgeR (Robinson et al., 2010). Pathway enrichment analysis for differentially expressed genes [DEGs; false discovery rate (FDR) < 0.01] was performed with g:Profiler (Raudvere et al., 2019) for Gene Ontology (GO) terms and Kyoto Encyclopedia of Genes and Genomes (KEGG) pathways (Kanehisa and Goto, 2000), and the full gene list for each comparison over all known rat genes was used as the statistical domain. Morpheus (https://software.broadinstitute.org/morpheus) was used to generate heat maps. Library preparation, sequencing, and data analysis were performed by the Gene Expression Center (Research Resource Identifier - RRID:SCR_017757), the DNA Sequencing Facility (RRID:SCR_017759), and the Bioinformatics Core Facility (RRID:SCR_017799), respectively, within the University of Wisconsin–Madison Biotechnology Center. Results were deposited in the Gene Expression Omnibus repository under accession number GSE278645.

### Protein isolation and cytokine analysis

Hippocampal tissues were collected from male and female rats at 8 or 12 weeks of age as described for RNA extraction. For analysis of cytokines, tissues from 8-week-old rats were homogenized with a GenoGrinder bead mill in RIPA buffer (Pierce, Thermo Fisher Scientific) with 1 mM Pefabloc SC (Sigma-Aldrich), and Complete Protease Inhibitor Cocktail (Roche, Sigma-Aldrich) at 150 mg tissue per milliliter. Samples were centrifuged at 20,000 g for 10 min at 4°C, supernatants were collected, and protein was quantified using the BCA assay (Pierce, Thermo Fisher Scientific). Homogenates were assayed using the Meso Scale Discovery (MSD) Proinflammatory Panel 2 (rat) V-PLEX kit to quantify IL1β, IL4, IL5, IL6, IL10, IL13, IFNγ, TNFα, and CXCL1 on a MESO QUICKPLEX SQ 120 multiplex cytokine plate reader within the Small Molecule Screening Facility at UW-Madison. For analysis of chemokines, tissues from 12-week-old rats were homogenized with a GenoGrinder bead mill in ProcartaPlex cell lysis buffer (Invitrogen, Thermo Fisher Scientific) with 1 mM Pefabloc SC and Complete Protease Inhibitor Cocktail at 200 mg tissue per milliliter. Samples were centrifuged at 16,000 g for 10 min at 4°C, supernatants collected, and protein was quantified using the BCA assay. Homogenates were assayed using a ProcartaPlex Rat Chemokine 8-plex Beads panel to quantify CXCL1, CXCL2, CXCL10, CCL2, CCL3, CCL7, and CCL11 on a Luminex MAGPIX Multiplex Analyzer plate reader within the Vision Research Core at UW-Madison. CXCL1 was below the limit of detection for the Luminex assay, and values from the MSD assay were reported.

### Protein isolation and western analysis

Hippocampal tissues were collected from male and female rats at 8 weeks of age as described for RNA extraction above. For analysis of postsynaptic proteins, tissues were homogenized with a GenoGrinder bead mill in 2% SDS, 50 mM Tris-HCl, pH 7.5, 5 mM EDTA, 1 mM Pefabloc SC (Sigma-Aldrich), and Complete Protease Inhibitor Cocktail (Roche, Sigma-Aldrich) at 50 mg tissue per milliliter, and protein was quantified using the BCA assay (Pierce, Thermo Fisher Scientific). Protein (5 µg/lane) was separated on 10% TGX Criterion gels (Bio-Rad) and transferred to Immobilon-IF PVDF membrane. After transfer (30 V 15 h, Criterion transfer system, 4°C), membranes were stained with REVERT Total Protein Stain and imaged on an Odyssey imager (LI-COR Biosciences) for quantification and normalization of proteins of interest. Membranes were then placed in SEA blocking buffer (Pierce, Thermo Fisher Scientific) before proceeding to incubations with primary antibodies against postsynaptic density protein 95 (PSD95; Cell Signaling 3450) or AMPA glutamate receptor subunit A1 (GluA1; Cell Signaling 13185) diluted 1:1,000 in TBS with 0.05% Tween 20 (TTBS) at 4°C overnight. Immunoblots were washed three times in TTBS, incubated with a secondary antibody (1:10,000 IRDye-800CW-conjugated goat anti-rabbit, LI-COR 925-32211) at room temperature for 2 h, washed again with TTBS followed by PBS, and allowed to dry before analysis with an Odyssey imager.

For vesicular presynaptic proteins and gephyrin, hippocampi were homogenized on ice in Syn-PER synaptic protein extraction reagent (Pierce, Thermo Fisher Scientific) with 1 mM Pefabloc SC and Complete Protease Inhibitor Cocktail at 10 mg/ml using a Potter-Elvehjem tissue homogenizer (900 rpm Teflon pestle in a glass tube). Protein from the synaptic fraction (P2 per manufacturer's protocol) was quantified with the BCA assay and 1 µg loaded (without heating) on 10% separating gels and transferred to nitrocellulose at 100 V for 70 min. Membranes were blocked in TTBS with 5% BSA before proceeding to protein staining (as above) and incubating with primary antibodies against synaptic vesicle glycoprotein 2A (SV2A; Cell Signaling 66724), vesicular glutamate transporter 1 (VGluT1; Cell Signaling 47181), vesicular GABA transporter (VGAT; Synaptic Systems 131 004), or gephyrin (Proteintech 12681-1-AP) diluted 1:1,000 in TTBS overnight at 4°C. Immunoblots were washed three times in TTBS; incubated with peroxidase-conjugated goat anti-mouse, rabbit, or guinea pig secondary antibodies (Jackson ImmunoResearch Laboratories, 115-035-003, 111-035-003, 106-035-003, diluted 1:5,000) at room temperature for 2 h; and washed again with TTBS before developing with Western Lightning Plus chemiluminescent substrate (Perkin Elmer) and imaging with an Azure 500 system.

For mitochondrial proteins, hippocampi were homogenized on ice in RIPA buffer (Pierce, Thermo Fisher Scientific) with 1 mM Pefabloc SC and Complete Protease Inhibitor Cocktail at 10 mg/ml using a Potter-Elvehjem tissue homogenizer (900 rpm). Homogenates were centrifuged at 12,000 g, protein from the supernatant was quantified with the BCA assay, and 8 µg was loaded (without heating) on a 12% separating gel and transferred to PVDF in Bjerrum Schafer-Nielsen transfer buffer at 100 V for 70 min. Immunolabeling was performed using the Total OXPHOS Rodent Western Blot Antibody Cocktail (Abcam ab110413) as described for synaptic proteins.

### Immunolabeling and stereology

Animals were deeply anesthetized with isoflurane and transcardially perfused with saline followed by 4% paraformaldehyde. Brains were removed and postfixed in paraformaldehyde before cryoprotecting in 30% sucrose and collecting 40 µm coronal sections on a sliding microtome. For stereology, every sixth section (240 µm interval) starting at the rostral extent of the hippocampus (∼−1.4 mm from the bregma) was immunolabeled for NeuN to count neuronal nuclei in the pyramidal layers and the granule cell layer of the DG. Pyramidal cell nuclei in CA1 and CA3 and dentate granule cell nuclei were outlined separately using Stereo Investigator (MBF Bioscience) for both hippocampi for a total of six contours per section when all regions were present. Cell counting was performed in both hemispheres on sections anterior to the posterior commissure (∼−4.5 bregma). For rats at 8 weeks of age, the sampling grid was 180 × 180 µm, the counting frame was 20 × 20 µm, and the disector height was 25 µm. For rats at 3 weeks, the sampling grid was 150 × 150 µm, the counting frame was 20 × 20 µm, and the disector height was 20 µm. Even numbers of males and females were analyzed in both age groups (*N* = 3), and data were combined for the final analysis after confirming there was no significant effect of sex. The Gundersen coefficient was <0.1 (*m* = 1) for all samples, and the total cell population was estimated for the dorsal hippocampus using the mean section thickness.

### Hippocampal long-term potentiation (LTP)

#### Slice preparation

Coronal hippocampal slices (400 µm) were prepared from 16-week-old rats. The rats were deeply anesthetized using isoflurane and decapitated. The brain was removed from the skull within 60 s of decapitation and immediately placed in a slice preparation solution containing the following (in mM): 124 NaCl, 1.25 NaH_2_PO_4_, 3 KCl, 25 NaHCO_3_, 10 glucose, 1 sodium ascorbate, 3 kynurenic acid, 3.6 MgSO_4_, and 0.8 CaCl_2_ and saturated with carbogen (95% O_2_/5% CO_2_). Brain slices were cut with a vibratome (Model 7,000 smz2, Campden Instruments) and transferred into a submerged incubation chamber containing slice recovery solution of the following (in mM): 124 NaCl, 3 KCl, 1.25 NaH_2_PO_4_, 25 NaHCO_3_, 15 glucose, 0.8 sodium ascorbate, 1.3 MgSO_4_, and 2.5 CaCl_2_. Slices were recovered at 33°C for 30 min followed by a room temperature recovery for 60 min. Both the preparation and recovery solutions were buffered to pH 7.3 with an osmolality between 294 and 297 osmol/kgH_2_O.

#### LTP

Recordings were taken from coronal slices in a submersion recording chamber perfused with the carbogenated recovery solution at 3.0 ml/min at 30°C using fire-polished borosilicate glass recording pipettes filled with 1 M NaCl (3–5 MΩ), and evoked responses were generated using Pt/Ir concentric bipolar stimulating electrodes. WinLTP software (v2.3, Bristol University) was used for stimulation and recording. LTP was induced using three theta burst trains consisting of 40 stimuli grouped into 10 bursts of four stimuli each at 100 Hz with burst delivered at 5 Hz. Potentiation was defined as the mean field excitatory postsynaptic potential (fEPSP) slope during the last 10 min of the 60 min LTP recording compared with the average fEPSP slope during the 10 min period preceding the theta burst stimulus.

### Novel object recognition (NOR)

A total of six litters were used to generate two cohorts of R237H and WT littermates (*N* = 24 with 12 males and 12 females per genotype) for the NOR test. Animals were tested at 16 weeks of age. Testing took place in a black Plexiglas arena (60 × 60 × 50 cm) over 2 consecutive days. On Day 1, rats were placed in an empty arena for 30 min to habituate them to the apparatus and then returned to their home cage. On Day 2, the familiarization phase, they were returned to the empty arena and allowed another 30 min of habituation. Two identical objects were then placed into the arena with the animals, and they were allowed 10 min to freely explore the two objects. Following exploration of the now familiar objects, rats were removed from the arena and isolated in Plexiglas holding cages for at least 60 min. One of the two objects was then replaced with a novel object. Rats were returned to the testing arena and allowed 5 min to freely explore the familiar and novel objects. Time spent exploring the objects was determined and used to generate a discrimination index (DI; time exploring novel object/total time exploring both novel and familiar objects) as a measure of NOR and memory for previously seen objects (Ennaceur and Delacour, 1988; Winters et al., 2008; Kinnavane et al., 2015). A glass jar and plastic cone were used as the stimulus objects. Right/left object position and designation of jar/cone as novel/familiar objects were counterbalanced across experimental groups. All trials were tracked using EthoVision software (EthoVision v.13.0, Noldus Information Technology), and time with the familiar and novel object was hand-scored by the investigator.

### Barnes maze

A total of 13 litters were used to generate two cohorts of R237H (*N* = 24; 11 males, 13 females) and WT (*N* = 30; 14 males, 16 females) littermates for the Barnes maze test (separate cohorts from those tested for NOR). Animals were tested at 16 weeks of age. The Barnes maze consists of a circular black Plexiglas test arena (170 cm in diameter) with 20 circular holes (12 cm in diameter) around the perimeter. One hole is designated as the escape hole and has a dark goal box underneath that the rat can enter. Rats were tested for a total of 6 d, with 1 habituation day and 5 training days. The maze was surrounded by black curtains and visual cues (e.g., white square, circle, triangle, and “N” shape) were attached to the curtain and present throughout training. On habituation day, the rats were placed on the arena for 10 min with ambient light (30 lux), with no curtain cues or goal box present. They were then returned to their home cage. The following day, rats were placed under a black Plexiglas box (∼20 × 20 × 15 cm) in the center of the arena for 60 s. Bright lights of ∼1,000 lux and ∼60 dB of white noise were then turned on. After 60 s, the rat was released from the box and was given 90 s to find the escape hole. If the rat did not find the escape hole, the investigator led the rat to the hole. In both cases, lights and sound were turned off when the rat entered the escape hole. The rat then remained in the escape hole for 15 s before starting the next trial. Each rat had four trials per day over the 5 d testing period. During each trial, the investigator recorded the latency to reach the escape hole (whether or not they escaped), total distance traveled, and search strategy used on the maze (random, serial, or direct) as described previously (Fedor et al., 2010; Pitts, 2018). Briefly, spatial strategies included a direct (the rat goes directly to the escape hole with no incorrect holes chosen) or serial search around the perimeter of the maze in either the clockwise or counterclockwise direction visiting two or more holes. All other patterns of movement on the maze were classified as random. Latency to locate the escape hole and spatial search strategy were analyzed.

### Statistical analyses

Data resulting from molecular, histological, and physiological analyses were analyzed using GraphPad Prism. For data sets with a single variable, an unpaired two-tailed *t* test was used to determine significance. For data sets with two variables, a two-way ANOVA was used to determine significance. All data in graphs represent means ± standard deviation.

Behavioral data were analyzed using SPSS statistical package (IBM SPSS Statistics, Version 28.0.0.0.). For the NOR test, time spent exploring the familiar and novel objects was analyzed by a two-way repeated measures ANOVA. A DI was calculated as the ratio of time spent exploring the novel object divided by the total time spent exploring the novel and familiar objects (i.e., novel/novel + familiar). Using this index, a lack of preference for novel versus familiar objects yields a DI of 0.5. A one-sample *t* test was used to determine whether the DI differed significantly from 0.5 and was used as an index of the strength of recognition memory (Brown and Aggleton, 2001). The DI corresponds to *d*′, one of the primary measures of recognition memory in humans (Sivakumaran et al., 2018). Latency to find the escape hole in the Barnes maze was analyzed by a mixed-effects model with Šídák's multiple-comparisons post-test. Search strategy (direct, serial, and random, as described in Barnes maze methods above) used to locate the escape hole was analyzed by chi-square.

**Figure 1.**
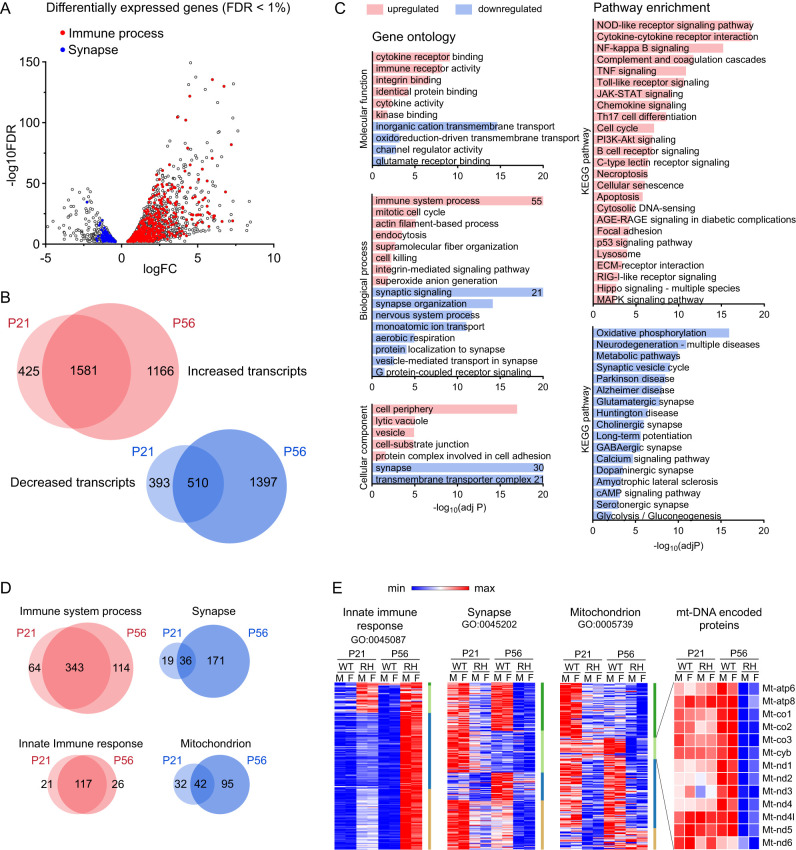
Neurodegenerative profile of hippocampal transcripts in the R237H rat model of AxD. ***A***, Volcano plot showing fold change (log_2_FC) values versus the FDR (log_10_FDR) for DEGs in 8-week-old male R237H rats compared with WT (FDR < 0.01). Genes related to the immune system process (GO:0002376) are highlighted in red, and those related to synapses (GO:0045202) are in blue. ***B***, Venn diagrams showing overlap in the number of DEG (increased and decreased) at P21 and P56. ***C***, Enrichment analysis for GO terms and KEGG pathways for DEG (FDR < 0.01) in male rats at 8 weeks of age (*N* = 4). For pathways with a −log_10_ adjusted *p* value greater than 20, values are indicated within the graph. ***D***, Venn diagrams showing overlap in the number of DEG related to different GO terms: immune system process (GO:0002376), innate immune response (GO:0045087), synapse (GO:0045202), and mitochondrion (GO:0005739) at P21 and P56. ***E***, Heat maps for DEG between WT and R237H (RH) rats within male (M) or female (F) groups, at P21 or P56 matched to different GO term gene lists as indicated. Average transcripts per million values were transformed to the color-coded minimum and maximum scale for each gene and K-means clustering used to highlight different patterns in developmental expression versus genotype-related changes. For each group, *N* = 4, except for WT females at P21, *N* = 3, and R237H females at P21, *N* = 5. Extended Data Figures 1-1–1-4 support this figure.

Data were examined for missing values and outliers. Outliers were identified from *z*-transformed data as those exceeding ±3.29. Missing data and outliers were replaced by multiple imputation (SPSS). Levene's test for homogeneity of variance was carried out, and variables violating assumptions of homogeneity were analyzed via the Mann–Whitney *U* nonparametric statistic. All tests were two-tailed with the minimum probability to establish statistical significance set at *p* < 0.05. All data in graphs represent means ± standard error of the mean ( $\bar{X}$ ± SE). The minimum probability for statistical significance was set at *p* < 0.05. Experimenters were blinded to genotype throughout behavioral testing, although the smaller size of R237H versus WT rats was readily apparent from ∼5 weeks of age, and this difference continued through the experiments (Fig. 1*F*, Hagemann et al., 2021).

## Results

### Neurodegenerative profile of hippocampal transcripts in R237H rat model of AxD

The R237H rat model of AxD exhibits extensive GFAP accumulation and aggregation throughout the CNS, with a particularly high pathological burden in the hippocampus (Hagemann et al., 2021). Given the role of the hippocampus in learning and memory, we wanted to understand the impact of reactive glia and astrocyte dysfunction on neurons and cognitive function and began with bulk RNA sequencing for differential gene expression analysis. To distinguish initiating events from chronic disease, we collected the hippocampus at 3 weeks of age (P21), when CNS pathology is apparent but R237H rats are otherwise presymptomatic, and at 8 weeks of age (P56), when pathology is significantly worse and the rats are severely affected with motor impairment and increased mortality. Both male and female R237H rats were analyzed with WT littermate controls, and gene expression comparisons were made between genotypes within age groups. In both age groups, elevated transcripts in the R237H rat hippocampus (FDR < 0.01) were dominated by immune-related genes, which demonstrated the largest fold changes. Those that were decreased encoded proteins related to synaptic and metabolic function (Fig. 1*A*, P56 males shown; Extended Data Figs. 1-1–1-4). Given the large number of genes affected (Fig. 1*B*), those that were upregulated were analyzed for pathway enrichment separately from genes that were downregulated (2,747 increased, 1,907 decreased, FDR < 0.01 in P56 male R237H rats). Enrichment results for GO terms were filtered using a two-stage algorithm within g:Profiler to group significant terms into subontologies and identify functional drivers within these contexts (Raudvere et al., 2019). At 8 weeks of age when disease is most severe, molecular functions of genes with increased expression were related to cytokine and immune receptor binding and activity, integrin binding, and downstream effects such as protein kinase binding (Fig. 1*C*, males shown; Extended Data Figs. 1-1, 1-2). Enriched biological processes again included immune system components and integrin-mediated signaling pathways as related drivers. Actions involved in reactive gliosis, including cytoskeletal and extracellular matrix reorganization (e.g., actin filament and supramolecular fiber organization) and pathways related to the cell cycle, were also highlighted as driving processes. Enriched cellular components included lytic vacuoles (lysosomes), vesicles (including endosomes), and cell adhesion terms such as focal adhesions, integrins, and associated signaling molecules (Fig. 1*C*; Extended Data Figs. 1-1, 1-2).

10.1523/ENEURO.0504-24.2025.f1-1 Figure 1-1 DEG for male rats at P56. Download Figure 1-1, XLS file.

**Figure 2.**
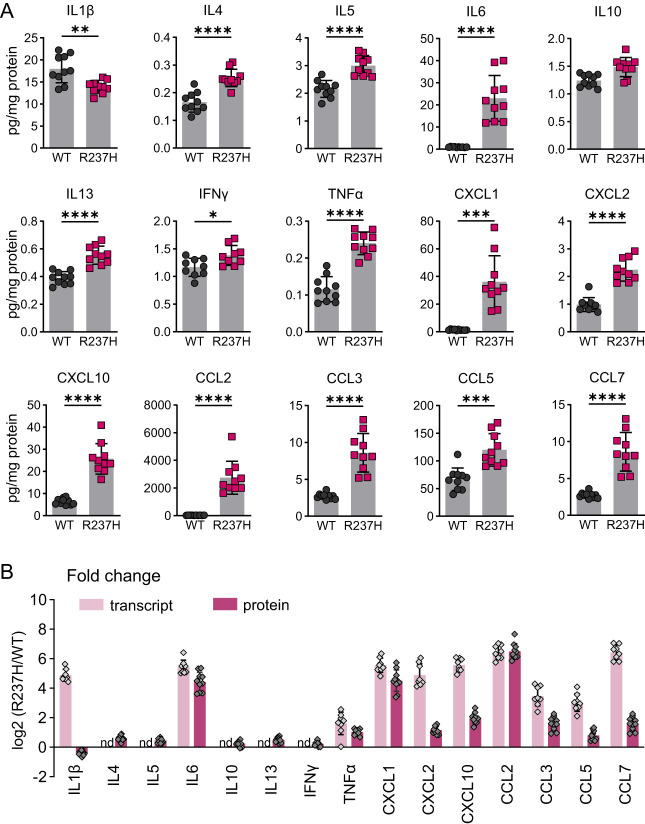
Neuroinflammatory response to GFAP mutation in AxD model rats. ***A***, Cytokines and chemokines were quantified by ELISA in adult R237H rats and WT littermate controls. Interleukins, IFNγ, TNFα, and CXCL1 were analyzed at 8 weeks, and the remaining chemokines were analyzed at 12 weeks of age. Data were analyzed with a two-tailed unpaired *t* test with Welch's correction, *p < 0.05, **p < 0.01, ***p < 0.001, ****p < 0.0001, *N* = 5 males, and 5 females for each group. Error bars are standard deviation. ***B***, Comparison of R237H with WT fold change for transcript and protein expression for the same cytokines and chemokines shown in ***A*** (nd indicates transcript not detected).

10.1523/ENEURO.0504-24.2025.f1-2 Figure 1-2 DEG for female rats at P56. Download Figure 1-2, XLS file.

KEGG pathway enrichment analysis demonstrated activation of NFκB and JAK-STAT signaling (Fig. 1*C*), consistent with our previous report showing both NFκB and STAT3 are activated in the R237H rat hippocampus (Hagemann et al., 2021). Transcripts related to cellular senescence and p53 signaling, including *Cdnk1a* (p21WAF), cyclins and checkpoint kinases, GADD45 genes, and Tp53 itself, were also significantly enriched, corroborating our recent work showing astrocyte senescence in the rat as well as other models of AxD (Wang et al., 2022). Pathways associated with an innate immune response were highly enriched, and a response to danger-/pathogen-associated molecular patterns (DAMPs) through NOD-like, Toll-like, and C-type lectin receptor signaling, cytosolic DNA sensing, and RIG-1-like receptor signaling could further contribute to astrocyte senescence and the associated secretory phenotype (Li and Chen, 2018). It is noteworthy that lymphocyte-related pathways are enriched, and although these pathways overlap with other immune-related categories, T-cell infiltration has been documented in AxD (Olabarria et al., 2015; Boyd et al., 2021). Comparisons between age groups showed that a majority of elevated transcripts were increased at both 3 and 8 weeks, with enrichment for most of the same immune-related pathways in each group (Fig. 1*D,E*). A heat map of significantly increased innate immune-related transcripts shows a subset of genes with comparable increases at both presymptomatic (P21) and severe stages (P56), while other transcripts demonstrate a progressive increase as the animals age (Fig. 1*D*; Extended Data Figs. 1-1–1-4). Many of the increased transcripts with an FDR < 0.01 at P21 and not at P56 (425 transcripts, Fig. 1*B*) were elevated at P56 when an FDR < 0.05 was considered (97/425). Of the 425 unique transcripts elevated at P21, 101 were downregulated in R237H rats at P56 compared with R237H rats at P21; however, there were no common pathways associated with these genes.

Transcripts that were decreased in the R237H rat consisted of genes related to synapses and metabolism and depicted a neurodegenerative profile (Fig. 1*A*). GO term enrichment analysis for molecular function at 8 weeks of age showed decreased expression of inorganic cation transmembrane transporters (proton, metal ion, and potassium transporters), channel regulators, and proteins involved in glutamate receptor binding. Synaptic signaling and synaptic organization drove biological processes, with all terms relating to synaptic function including G-protein-coupled receptor signaling, monoatomic ion transport (potassium, calcium, and metal ion transport), and aerobic respiration (mitochondrial function). Enriched cellular components also consisted of synapse-related terms that included mitochondrial membranes and the respiratory chain complex. Many of these changes were not yet apparent at 3 weeks of age in asymptomatic rats (Fig. 1*D,E*; Extended Data Figs. 1-3, 1-4). Enriched GO terms in rats at P21 included monoatomic ion transport and nervous system development as drivers of molecular function and biological process, respectively, but only cellular component terms showed a significant enrichment of synapse-related compartments (Fig. 1*D,E*; Extended Data Figs. 1-3, 1-4).

**Figure 3.**
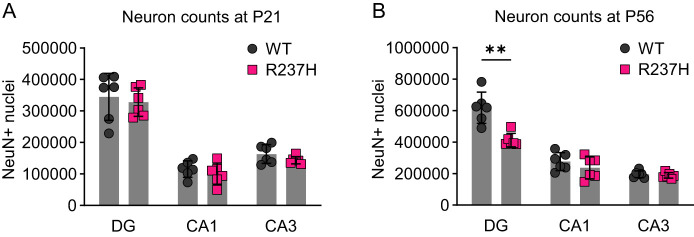
Regional differences in hippocampal neuron numbers in R237H rats compared with WT. Stereological counting of hippocampal neurons labeled with NeuN at P21 (***A***) and P56 (***B***). NeuN-labeled nuclei were counted in the DG granule cell layer and CA1 and CA3 pyramidal cell layers throughout the dorsal hippocampus (anterior boundary to posterior commissure). Data were analyzed by two-way ANOVA and Šídák's multiple-comparisons test (**p = 0.0073, *N* = 6 per group consisting of 3 males and 3 females). There was no significant effect of sex when sex was included as a variable in a three-way ANOVA.

10.1523/ENEURO.0504-24.2025.f1-3 Figure 1-3 DEG for male rats at P21. Download Figure 1-3, XLS file.

**Figure 4.**
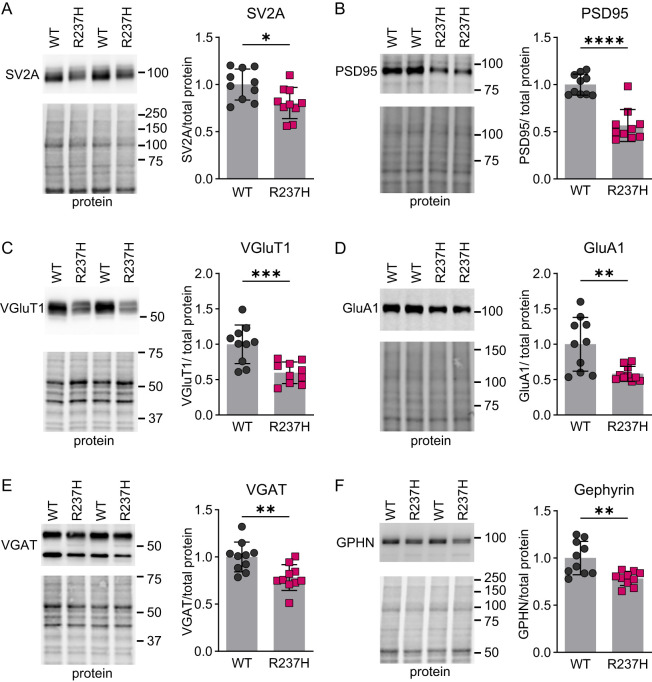
Changes in synaptic protein expression in R237H rats. ***A***, ***B***, Western analysis of pre- and postsynaptic proteins SV2A and PSD95, respectively, in the hippocampus. Representative images are shown for immunoblots and corresponding protein stain from the same region of the gel with molecular weight marker positions indicated on the right. Values for each protein are normalized to total protein and expressed as a fraction of the WT group. ***C***, ***D***, Western analysis of excitatory pre- and postsynaptic markers VGluT1 and GluA1 (***E***, ***F***) and inhibitory markers VGAT and gephyrin. Data were analyzed with a two-tailed unpaired *t* test, *p < 0.05, **p < 0.01, ***p < 0.001, ****p < 0.0001, *N* = 5 males, and 5 females at 8 weeks of age for each group.

10.1523/ENEURO.0504-24.2025.f1-4 Figure 1-4 DEG for female rats at P21. Download Figure 1-4, XLS file.

KEGG pathway enrichment analysis for transcripts that were decreased at 8 weeks of age in the R237H rat hippocampus again highlighted a neurodegenerative profile and included “pathways of neurodegeneration—multiple diseases,” as well as Alzheimer’s, Parkinson’s, and Huntington’s diseases, and amyotrophic lateral sclerosis. Multiple synaptic profiles, including those for glutamatergic, GABAergic, cholinergic, dopaminergic, and serotonergic synapses, showed decreased expression, as predicted by GO term analysis. Transcripts related to oxidative phosphorylation and mitochondrial function were highly enriched among downregulated genes, and genes related to calcium and cyclic AMP signaling also showed decreased expression (Fig. 1*C*). At 3 weeks of age, pathways of neurodegeneration were not significantly enriched in R237H rats. Comparisons of expression changes for synapse-related genes (GO:0045202) between age groups demonstrated expected developmental changes (within both genotypes) but a general trend for decreases in AxD model rats at both presymptomatic and severe disease stages (Fig. 1*D,E*). A higher number of genes related to mitochondrial function were downregulated at 8 weeks compared with 3 weeks, accounting for the shift to a neurodegenerative profile at the later age (Fig. 1*D,E*). Genes encoded by mitochondrial DNA were not downregulated at P21, but transcripts from all 13 protein encoding genes were decreased at P56 as well as many nuclear genes related to oxidative phosphorylation and respiration, suggesting an overall reduction of mitochondrial function at this severe stage of disease (Fig. 1*D*).

### Neuroinflammatory response to GFAP mutation in AxD model rats

We have previously demonstrated activation of NFκB and STAT3 in the R237H rat hippocampus and specifically nuclear localization of phosphorylated STAT3 (Tyr705) in astrocytes (Hagemann et al., 2021). Here, to determine whether elevation of immune-related transcripts translated into a neuroinflammatory microenvironment, we quantified cytokines and small chemokines in adult rat hippocampus by ELISA (Fig. 2*A*). In agreement with STAT3 activation and our previous report showing a senescence phenotype in AxD astrocytes (Wang et al., 2022), IL6 was markedly elevated (24-fold, *p* < 0.0001), and TNFα was also significantly increased (two-fold, *p* < 0.0001), similar to their transcription profile (Fig. 2*B*). Although IL1β transcript is elevated, protein was slightly decreased, and many of the other interleukins analyzed were near the lower limits of detection and only marginally elevated, including anti-inflammatory cytokines IL10 and IL4. It is possible that the assay used may not detect pro-IL1β or oxidized/reduced forms (per the manufacturer) or that heterogeneous nuclear ribonucleoproteins may affect translation (Sirenko et al., 2002; Zhao et al., 2012). Many small chemokines were highly elevated, including CXCL1 and CCL2, suggesting potential chemotactic effects on peripheral immune cells. CXCL10, an IFNγ-inducible chemokine, is also markedly elevated, although IFNγ is only marginally increased.

### Regional differences in hippocampal neuron numbers

The decrease in synapse-related gene expression observed in the R237H rat hippocampus (Fig. 1*C*) could reflect neuronal loss, and we have previously reported that granule cells in the hippocampal DG are reduced in number (Hagemann et al., 2021). To analyze both the DG and pyramidal cell layers, we counted pyramidal cells in CA1 and CA3 and granule cells in the DG of the dorsal hippocampus using stereological methods at 3 and 8 weeks of age. At 3 weeks of age, there were no differences in the regions analyzed; however, a reduction in granule cell numbers was confirmed in the DG at 8 weeks of age (Fig. 3). This is of particular interest in view of the involvement of the DG in the formation of precise memories for locations of objects (Lee and Jung, 2017). No change in pyramidal cell numbers was detected in CA1 or CA3 at 8 weeks (Fig. 3).

### Neurodegenerative changes in synaptic and mitochondrial proteins in R237H rat hippocampus

**Figure 5.**
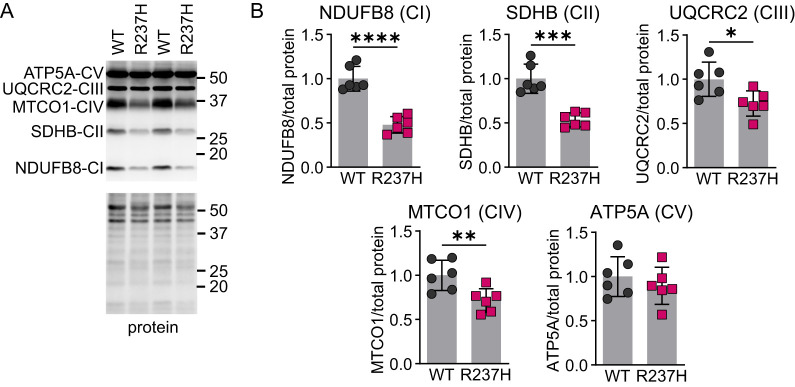
Changes in mitochondrial proteins in R237H rats. ***A***, ***B***, Western analysis of subunits of the oxidative phosphorylation complexes I–V in the rat hippocampus. ***A***, Representative images are shown for immunoblots and corresponding protein stain from the same region of the gel with molecular weight marker positions indicated on the right. ***B***, Values for each subunit are normalized to total protein and expressed as a fraction of the WT group. Data were analyzed with a two-tailed unpaired *t* test, *p < 0.05, **p < 0.01, ***p < 0.001, ****p < 0.0001, and *N* = 3 males and 3 females at 8 weeks of age for each group.

To further assess neuronal pathology and potentially synapse function, we quantified expression of synapse-related proteins in the hippocampus from R237H rats at 8 weeks of age. An initial assessment by western analysis showed decreases in both pre- and postsynaptic proteins including SV2A and PSD95, respectively (Fig. 4*A,B*). More specific analysis of excitatory synaptic markers VGluT1 and GluA1 also demonstrated decreases at the protein level (Fig. 4*C,D*). Inhibitory pre- and postsynaptic markers VGAT and gephyrin were also reduced (Fig. 4*E,F*). In addition, we analyzed a panel of mitochondrial proteins related to oxidative phosphorylation (Fig. 5) from complexes I to V (NDUFB8, SDHB, UQCRC2, MT-CO1, and ATP5A). All but complex V (ATP5A) showed a decrease in the R237H rat hippocampus at 8 weeks of age, including the mitochondrially encoded cytochrome C oxidase I (complexes III and IV).

### Deficits in hippocampal LTP in R237H rats

**Figure 6.**
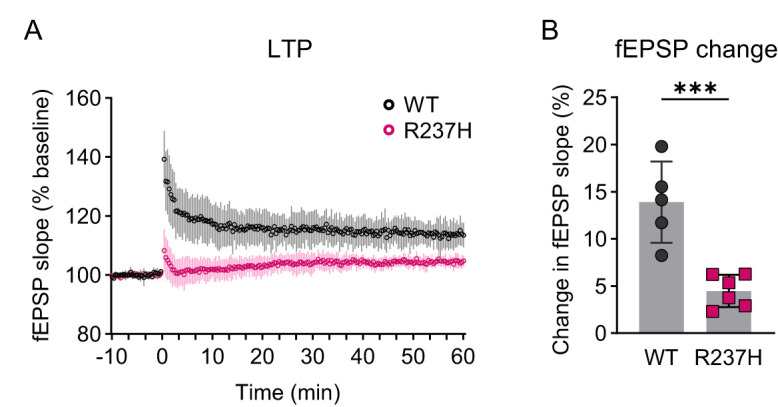
Deficits in hippocampal LTP in R237H rats. ***A***, Mean fEPSP slope for WT animals before and after a TBS paradigm with three trains of 10 bursts (4 × 100 Hz). ***B***, Comparison of potentiation, defined as the average mean fEPSP slope during the last 10 min compared with baseline. Data were analyzed with a two-tailed unpaired *t* test, ***p = 0.0008, and *N* = 5 WT (2 males, 3 females), and 6 R237H (3 males, 3 females) rats. Error bars = standard deviation.

To determine whether differences in expression of synaptic proteins translated into physiological deficits, we analyzed the R237H rat hippocampus for LTP in Schaffer collateral-CA1 synapses. To match the age at which we planned to test cognitive function, LTP was measured at 16 weeks, when R237H rats show improved motor performance and are past the critical survival period between 6 and 12 weeks of age when 15% of the animals die. Acute coronal slices including the dorsal hippocampus were collected from both male and female rats of each genotype and subjected to a theta burst stimulation (TBS) paradigm with three trains of 10 bursts (4 × 100 Hz). The fEPSP was measured for 10 min before and 1 h after TBS. Potentiation for each slice was defined as the mean fEPSP slope during the final 10 min compared with baseline (Fig. 6*A*). There was significantly less potentiation (*p* = 0.0008, *t* test) for R237H rats versus WT rats, confirming impaired synaptic plasticity at this age in the model (Fig. 6*B*).

### Deficits in NOR in R237H rats

**Figure 7.**
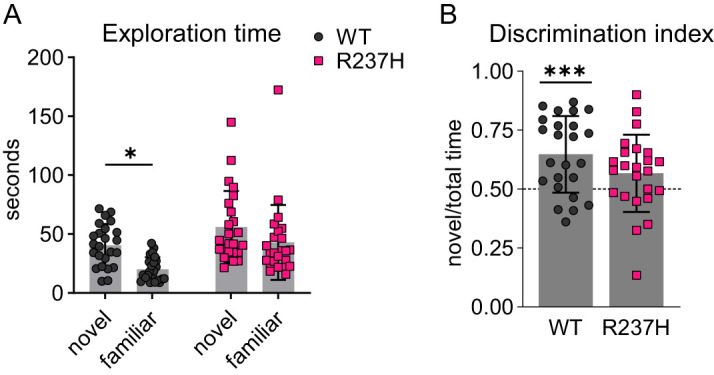
Impaired NOR performance in R237H rats compared with WT. ***A***, Time (seconds) exploring novel and familiar objects for WT and R237H rats in the NOR test. Both novelty and genotype were significant sources of variation (novelty = 9.5% of variation, *p* = 0.0033; genotype = 12.4% of variation, *p* = 0.0001; two-way repeated measures ANOVA with uncorrected Fisher's LSD post-tests, *p* = 0.0104). ***B***, DI as a measure of NOR performance for WT and R237H rats. A DI significantly greater than 0.5 (chance) indicates a preference for exploration of the novel object, whereas a DI that is not significantly different from 0.5 indicates impairment in recognition memory (one-sample *t* test, ***p = 0.0002). *N* = 24 WT (12 males, 12 females) and 24 R237H (12 males, 12 females) rats at 16 weeks of age for ***A*** and ***B***.

The NOR task was used as an initial test to evaluate recognition memory in the R237H rat. This test is based on the tendency of rats to spend more time exploring novel objects than exploring familiar objects (Ennaceur and Delacour, 1988). Current evidence supports a critical role for the perirhinal cortex and its projections to the hippocampus, including the DG, in object memory encoding, consolidation, and retrieval (Brown and Aggleton, 2001; Winters et al., 2008; Kinnavane et al., 2015). R237H rats and littermate controls were tested at 16 weeks of age. WT rats showed longer exploration of the novel object compared with the familiar object with a significant effect of genotype (two-way RM ANOVA, *p* = 0.0001), while R237H rats did not show a preference (Fig. 7*A*; data from one female R237H rat was excluded from analysis due to a lack of exploratory activity during the testing phase). A DI representing the time exploring the novel object as a fraction of the total time exploring both objects was calculated as an additional measure of NOR performance. DIs for recognition memory performance in male (*p = 0.018) and female WT (**p = 0.006) rats were significantly greater than 0.5 (i.e., chance) indicating memory for previously presented objects (Fig. 7*B*). In contrast, DIs were not significantly above chance for either male or female R237H rats.

### Impaired Barnes maze performance in R237H rats

**Figure 8.**
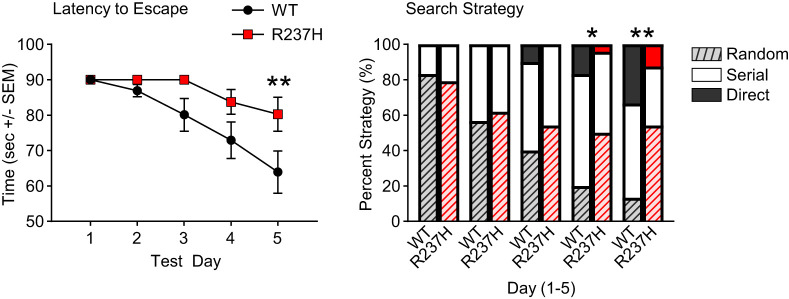
Barnes maze performance for WT and R237H rats. ***A***, Latency to escape during the first trials over 5 consecutive days of training (*p* = 0.018 for effect of genotype, mixed-effects model; **p < 0.01, Šídák's post-test, error bars = SEM). ***B***, Search strategy patterns (random, serial, and direct; see Materials and Methods section) used by rats to learn the location of the escape hole in the Barnes maze. The first trials for each of the 5 training days are shown for comparison between WT and R237H rats (chi-square; *p < 0.05 and **p < 0.01). *N* = 30 WT (14 males, 16 females), 24 R237H (11 males, 13 females), at 16 weeks of age for ***A*** and ***B***. Average latency to escape and search strategies for all four trials are shown in Extended Data Figure 8-1.

The Barnes maze is a behavioral test of spatial memory (Barnes, 1979). It shares similarities with the Morris water maze (Morris, 1984) in that it requires an animal to use distal environmental cues to locate a fixed escape location and has the advantage of not requiring stress associated with swimming. The goal of this task is for the rats to learn to seek shelter from bright lights by finding a dark escape hole. Male and female R237H rats and littermate controls were tested with four trials per day over 5 consecutive days at 16 weeks of age, and data were analyzed separately for each day as well as the average daily score across the four daily trials using a day-by-genotype-by-sex repeated measures ANOVA. Data for Trial 1 on each test day was used to assess spatial reference in the Barnes maze and proved to be the most reliable as rats tended to perform poorly over repeated trials each day once they had located the escape hole in Trial 1. Figure 8*A* shows the average latency (seconds) to find the escape hole in Trial 1 on each of the 5 testing days. As expected, there was a significant overall decrease in latency to find the escape hole (*F*_4,188_ = 9.80; *p* < 0.001) across test days. There was also a significant difference between genotypes (*F*_1,47_ = 4.74; *p* < 0.05). Sex differences and interactions were not statistically significant. Comparisons of daily escape latencies between WT and R237H rats showed that WT rats had shorter escape latencies on Test Day 5 compared with R237H rats (*F*_1,52_ = 4.22; *p* < 0.05). Escape latencies did not differ on Test Days 1–4. The three nominal search strategies used by the rats (random, serial, or direct) were also analyzed for Trial 1 on each test day, as well as the average percent use of each strategy over the four trials on each test day. As shown in Figure 8*B*, rats begin the task by using mainly a random search strategy but shift to serial, and then a direct spatial strategy over the course of training. By Test Days 4 and 5, WT rats were using a search pattern that differed significantly by chi-square analysis from R237H rats (*p* < 0.05 and *p* < 0.01, respectively) in that fewer WT rats were using a random search strategy, and more were using a direct spatial strategy. The same general pattern was found when all four daily trials were included in a chi-square analysis (Extended Data Fig. 8-1).

10.1523/ENEURO.0504-24.2025.f8-1 Figure 8-1 Barnes maze performance for wild type and R237H rats. (**A**) Average latency to escape for 4 trials daily over 5 consecutive days of training, (no significant effect of genotype, mixed-effect models; **p < 0.01, Sidak’s post-test, error bars = SEM). (**B**) Search strategy patterns (random, serial, direct; see Methods section) used by rats to learn the location of the escape hole in the Barnes maze. Percent strategy used for all trials over each of the 5 training days are shown for comparison between wild type (WT) and R237H rats (Chi Square; ***p<0.001). N = 30 WT, 24 R237H, both sexes at 16 weeks of age for A and B. Download Figure 8-1, TIF file.

## Discussion

Cognitive delays and deficits are frequent clinical features of AxD, particularly in early-onset cases, but relatively few publications provide detailed information about the cognitive phenotype. Most studies are single-case reports, presenting evidence for cognitive decline using a variety of tests including unspecified IQ tests, Wechsler Intelligence Scales for adults and children, and tests for dementia. More recent studies have provided additional information about cognitive function, including impaired visual and verbal memory, using different assessment tools. For example, memory problems were reported in three subjects with adult-onset AxD, including impairments in short-term memory and tests of object recall (Anis et al., 2023). In another case study, two of three adult-onset AxD patients showed impaired verbal and visual memory in the Montreal Cognitive Assessment test (Lichtenstein et al., 2017). In a study of four children with AxD, ages 5–15, verbal short-term memory, backward verbal short-term memory, and narrative microstructure were impaired, along with word recall (Zampini et al., 2023). Additional case studies of patients with infantile onset support the conclusion that cognitive impairments, and memory deficits in particular, are common features of AxD (Draghi et al., 2019; Kirsch et al., 2021), but a better understanding of the cognitive phenotype and its underlying mechanism is necessary for the development and testing of potential therapeutics to address this aspect of the disease.

Memory processes involve interactions among widespread regions of the brain, including the prefrontal cortex, amygdala, striatum, and hippocampus, among others (White and McDonald, 2002; Kesner, 2009), and astrocyte pathology throughout the CNS is likely to contribute to memory impairment found in AxD (Li et al., 2025). In this report, we sought to take advantage of the R237H rat model of AxD to determine the impact of reactive and dysfunctional astrocytes on hippocampal neurons, memory, and learning. Transcriptomic analysis of hippocampal gene expression indicated early activation of stress-related pathways in AxD model rats prior to the onset of overt clinical phenotypes. Pathways related to immune processes were activated at 3 weeks of age and then intensified as disease progressed, similar to our previous transcriptomic studies in an early mouse model of AxD (Hagemann et al., 2005). Genes related to metabolism, particularly those involved in lipid and cholesterol biosynthesis, were downregulated at this early stage, and a subset of synaptic and neurodevelopmental transcripts were also decreased. The decline in synapse-related gene expression became more pronounced with disease progression with multiple neuronal subtypes being implicated, and pathways involved in oxidative phosphorylation and mitochondrial function were also decreased, depicting a general profile of neurodegeneration. Given the essential role of astrocytes in maintaining CNS homeostasis and that AxD is a primary disorder of astrocytes, these results suggest that astrocyte dysfunction may also be a driving factor in other neurodegenerative diseases.

We have recently reported that a subpopulation of astrocytes in AxD display a senescent phenotype, as demonstrated in multiple models, including the rat, and in patients with the disease (Wang et al., 2022). In addition, we have shown that gene expression profiles from our mouse models of AxD are highly congruent with a transcriptomic portrait of human Alzheimer's disease, second only to the Alzheimer's APP/PSEN1 mouse in comparison with large-scale gene expression data from over 500 animal models (Gammie et al., 2024). These results further suggest that chronic gliosis and astrocyte dysfunction contribute to cognitive impairment in both Alzheimer's disease and AxD, and early reports described AxD as a developmental form of Alzheimer's based on the marked oxidative stress response (Castellani et al., 1999). Here, we find an overlap between these two conditions in both the stress response and in the loss of markers for synaptic and mitochondrial functions. Whether metabolic changes occur in neurons, astrocytes, or multiple cell types is not clear, but studies with induced pluripotent stem cell-derived astrocytes from patients with AxD also demonstrate shifts in glycolysis and oxidative phosphorylation (Jones et al., 2018). Intermediate filaments have direct and indirect interactions with mitochondria (Schwarz and Leube, 2016), and GFAP mutations have been linked to lipoxidation and oxidative stress (Viedma-Poyatos et al., 2022). Mitochondrial dysfunction may also contribute to a cell-intrinsic innate immune response (Banoth and Cassel, 2018; Newman and Shadel, 2023), and future studies on the role of lipid metabolism and mitochondrial function will be important in understanding how astrocytes contribute to the neurodegenerative profile (Cali et al., 2024).

Immune-related responses are prominent at an early age in rodent models of AxD (Hagemann et al., 2005; Olabarria et al., 2015), and although neuroinflammation precedes severe cognitive deficits in neurodegenerative disorders of aging such as Alzheimer's disease, effects on mild cognitive impairment in early stages of disease are not clear (Heneka et al., 2015). Microglia play a pivotal role in recognizing amyloid β as a DAMP and triggering innate immune signaling, and although reactive astrocytes contribute to the inflammatory milieu, genetic risk factors such as TREM2 and CD33 variants implicate microglia as key players in Alzheimer's disease. In AxD, the primary insult is in astrocytes, but many of the same genes and pathways are activated in both diseases. Astrocyte-specific transcription profiling in AxD model mice at postnatal day 14, an early stage in GFAP accumulation and aggregation, shows a similar elevation in innate immune-related transcripts (Gammie et al., 2024), and our recent study analyzing the role of STAT3 in GFAP elevation and the astrocyte stress response has further indicated that the immune response is initiated in astrocytes (Hagemann et al., 2023). Nevertheless, microglia are reactive and in close association with astrocytes in AxD (Olabarria et al., 2015; Hagemann et al., 2021), and innate immune pathways are likely activated in both cell types. Future studies will include single-cell analysis to follow the trajectory of astrocyte pathology within different subtypes and noncell autonomous effects on microglia, neurons, and oligodendrocytes over time (Srinivasan et al., 2016; Mathys et al., 2019; Habib et al., 2020; Liddelow et al., 2020; Allen et al., 2022; Sadick et al., 2022; Mathys et al., 2024; O'Dea and Hasel, 2024).

The importance of the hippocampus for memory is well established (Scoville and Milner, 1957; Squire and Zola-Morgan, 1991), but the specific contributions of hippocampal subregions (e.g., DG, CA1, and CA3) and adjacent structures (e.g., perirhinal and entorhinal cortex) to explicit aspects of memory are still not well understood (Hainmueller and Bartos, 2020). To assess whether reduced expression of synaptic markers reflect neuronal loss in R237H rats, we quantified granule cells in the DG and pyramidal cells in CA1 and CA3 and found no change at 3 weeks but reduced numbers of granule cells at 8 weeks of age. We have previously shown that AxD model mice and rats exhibit deficits in adult neurogenesis, with virtually no detectable doublecortin-expressing neurons (Hagemann et al., 2013; 2021), which may contribute to the differences in cell number observed in the DG. Further analysis of synaptic proteins demonstrated a decrease in pre- and postsynaptic markers for both excitatory and inhibitory synapses, suggesting that neuronal deficits are not selective and supporting our transcriptomic analysis showing downregulation of several neurotransmitter signaling pathways including those for glutamatergic and GABAergic synapses. Importantly, we demonstrate that neuronal deficits translate into functional deficits, both physiologically with reduced hippocampal LTP and synaptic plasticity and behaviorally with impaired performance in tests of learning and memory. These findings contrast with earlier work showing increased hippocampal LTP and plasticity in GFAP-null mice (McCall et al., 1996) and further demonstrate the toxic gain of function caused by GFAP mutation.

Given the many differences we observed in immune factors, metabolic pathways, and synaptic proteins, and their potential effects on neurodevelopment, synaptic plasticity, and cognitive functions, we cannot attribute a single cause for impaired LTP and cognitive deficits in the R237H rat (Li et al., 2025). Astrocytes are intricately associated with synapses and respond to many of the same neurotransmitters via G-protein-coupled receptor activation and intracellular calcium mobilization (Durkee and Araque, 2019), and previous studies have shown that Ca^2+^-dependent astrocyte release of d-serine is necessary for NMDA receptor activation and LTP induction (Henneberger et al., 2010; Adamsky et al., 2018; Ahrens et al., 2024). A recent report has further demonstrated activation of c-Fos and increased Ca^2+^ activity in hippocampal astrocytes associated with engram neurons during contextual fear conditioning, and astrocyte-specific knock-out of c-Fos reduces Ca^2+^ activity, impairs learning, and diminishes hippocampal LTP (Williamson et al., 2025). Astrocytes in mouse models of AxD (Saito et al., 2018) and induced pluripotent stem cell-derived astrocytes from patients with the disease (Jones et al., 2018) demonstrate aberrant Ca^2+^ waves, and the transcriptomic analysis reported here shows reduced expression of genes related to calcium signaling. The marked reduction in LTP in the R237H rat model provides an opportunity for future studies to determine the degree to which loss of normal astrocyte function contributes to reduced synaptic plasticity in AxD.

Several overlapping memory processes have been proposed for the DG, including novelty detection (Hunsaker et al., 2008), recognition memory (Jiang et al., 2023), pattern separation (Gilbert et al., 2001), pattern completion (Nakashiba et al., 2012), binding of information to spatial contexts (Lee and Jung, 2017), and working memory (Sasaki et al., 2018). Given the crucial role of the hippocampus in the formation and recall of memories for objects, places, and events (Lisman et al., 2017), abnormalities in the DG likely contribute to deficits in both NOR and the Barnes maze, as observed in AxD model rats. Computational modeling suggests that dentate granule cells perform pattern separation on spatial representations arriving from the entorhinal cortex (Yassa and Stark, 2011; Kesner and Rolls, 2015), and recent optogenetic studies have shown that the perforant pathway, and more specifically projections from the medial entorhinal cortex layer II to the dentate, is an essential memory circuit for visually guided navigation tasks such as the Barnes maze (Qin et al., 2018). Given the role of the perforant pathway in memory impairment in Alzheimer's disease (Hyman et al., 1987; Harris et al., 2010), astrocyte pathology and neuronal deficits in the entorhinal cortex may also be worthy of future investigation in AxD.

In this report, we show that in addition to motor and myelin deficits (Hagemann et al., 2021), the rat model of AxD demonstrates neuronal and cognitive impairment as clinically relevant phenotypes. Future studies can take advantage of the model to better understand astrocyte dysfunction in AxD and neurodegenerative disease more generally.

## Synthesis

Reviewing Editor: Selva Baltan, Oregon Health &Science University Department of Surgery Decisions are customarily a result of the Reviewing Editor and the peer reviewers coming together and discussing their recommendations until a consensus is reached. When revisions are invited, a fact-based synthesis statement explaining their decision and outlining what is needed to prepare a revision will be listed below. The following reviewer(s) agreed to reveal their identity: James Goldman. Note: If this manuscript was transferred from JNeurosci and a decision was made to accept the manuscript without peer review, a brief statement to this effect will instead be what is listed below.

Please follow the reviewer instructions for the required revision.

## Author Response

January 28, 2025 Dear Dr. Baltan and reviewers, Thank you for your thoughtful response and suggestions for our manuscript. We hope our revisions adequately address concerns shared by the reviewers. Answers to your questions and an outline of changes to the manuscript are detailed in the point-by-point response below.

Many thanks for your time and effort.

----- Synthesis of Reviews (from the Editor):

Berman et al. have investigated a rat model of Alexander Disease (AxD) with particular attention of changes in cognitive deficits and synaptic changes. The analysis results in new insights into how astrocyte dysfunction produces behavioral disorders. The hippocampal RNA analyses point out important changes in gene expression having to do with immune function, synapses, and metabolic activity. The study nicely describes important gene expression changes and behavioral changes, although it is still difficult to ascertain causal relationships.

Comments:

1. Please specify exactly what hippocampal tissues were taken - that is, did they include para-hippocampal gyrus, including entorhinal cortex, for eg.? Changes in the entorhinal cortex would result in synaptic changes in the hippocampus because of the axonal projection.

We have added the following details to the Methods section (p. 7, lines 112-115):

Hippocampus included Ammon's horn, the dentate gurus, and the subiculum. The alveus was used as a dorsal boundary to separate the hippocampus from subcortical white matter, and parahippocampal regions were removed from the ventral/lateral boundaries. Excess white matter from the fimbria and fornix were also removed.

2. The study would have more impact if some of the major changes in gene expression were validated anatomically at a protein level and the authors should be encouraged to do that. They might find interesting and important changes that appear in different areas of the hippocampus. One wonders, for eg. if there is a loss of glutamatergic or GABA-ergic synapses. The Western blots do show decreases in proteins, but that does not necessarily translate into synaptic loss. The loss of granule cells could possibly account for this. Or the astrocyte pathology could also account for this, in a more widespread way.

We also were interested in including such data, and imaged different pre- and post-synaptic marker pairs for colocalization analysis in CA1 stratum radiatum. We found the results to be variable, and after analyzing 6 rats per genotype, deduced that the number of animals needed to sufficiently power the method in an outbred strain was prohibitive. In addition, there was concern that hypertrophic astrocytes in AxD model animals displaced marker labeling and potentially skew quantification.

3. It is interesting that many of the upregulated genes were of an immunological nature. Did the authors look for peripheral immune cells or reactive microglia that express more of these molecules? Maybe they are coming from the astrocytes, as the authors suggest. Single cell analyses would help to determine this, but that's for another study.

We have previously reported that Iba1 immunolabeling shows an increase in hypertrophic microglia in most regions of the CNS (Hagemann et al., 2021), including hippocampus, and as noted by the reviewers, we include a paragraph in the Discussion on whether immune-related transcripts originate from microglia or astrocytes. We have clarified that microglia are reactive in the rat model in the Discussion (p. 25, line 545).

We also mention in the Results that T-cell infiltration has been documented in AxD (Olabarria et al., 2015; Boyd et al., 2021) (p. 16, lines 338-340), but we have not specifically looked for other peripheral immune cells in the parenchyma or subarachnoid and perivascular spaces. As suggested by the reviewers (and noted in the manuscript), a more comprehensive analysis with single cell RNAseq would be informative in determining the origin of the extensive list of immune-related transcripts.

4. Do the authors think that the myelin defects play a role in the transcriptomic or behavioral abnormalities? Since AxD has a large dys/de-myelination component, how could this affect what they find in this model? We have demonstrated myelin deficits in spinal cord and optic nerve (Hagemann et al., 2021), and white matter pathology likely plays a role in behavioral phenotypes in the model. However, we do not capture those changes in the transcriptomic analysis in hippocampus performed here. Transcripts for genes related to myelination such as Plp1, Mag, Myrf, and Olig2 are slightly elevated in R237H rat hippocampus and show marginal enrichment in gene ontology analysis. These changes could reflect shifts in oligodendrocyte lineages and myelin repair (Chen et al., 2021), but it would be highly speculative to suggest without additional evidence. It is also possible that myelinated tracts from other brain regions to the hippocampus, such as perforant pathway fibers, are affected by myelin deficits, leading to synaptic loss or degeneration (Nasrabady et al., 2018), but as indicated above, we did not analyze parahippocampal regions and it remains to be determined whether there are deficits in these structures or their hippocampal projections. This is an important question, but the data in this report do not allow any substantive conclusions.

5. The changes in LTP are particularly intriguing. Given how much is known about LTP, which synapses are involved, which transmitter systems are involved, can the authors be more specific about LTP, more than simply writing that they demonstrate that "neuronal deficits translate into functional deficits"? What neuronal deficits translate into functional deficits? Given the many differences we observed in immunologic factors, metabolic pathways, and synaptic proteins, and their potential effects on neurodevelopment, synaptic plasticity, and cognitive functions, we cannot say which are most likely to cause impaired LTP and cognitive deficits. Nevertheless, the strong phenotype in the R237H rat provides an opportunity for future studies of causation.

To address the reviewers' point, we have added a paragraph focused on the role of astrocyte activation in LTP and the potential effects of changes in intracellular astrocyte calcium signaling, as reflected in the transcriptomic data and previously reported in the literature for AxD astrocytes (p. 26, lines 569-584).

6. Since the behavioral tests do involve memory functions, they may not necessarily involve only the hippocampus, and given that the astrocyte dysfunction is much more widespread than the hippocampus, the authors should take a broad approach in correlating behavior with region.

In this report, we focused on hippocampus due to the high levels of GFAP pathology compared with other brain regions such as cortex. Learning and memory in the novel object recognition test and Barnes maze are commonly associated with hippocampal function and are particularly relevant for deficits in adult neurogenesis. We agree that other brain regions (e.g. prefrontal cortex) could be functionally impacted, but to tease out which regions contribute to spatial memory, working memory, and the overall cognitive phenotype will require additional testing and experiments (e.g. Y-maze, variations of the Barnes maze, others).

We have added a statement to the Discussion to acknowledge that other brain regions are likely affected (p. 23, lines 494-497).

7. The discussion of AxD vs. neurodegenerative diseases is interesting, but can be shortened because some of it is very general, rather than specific.

Deleted lines 508-512 (p. 23) from Discussion.

Boyd MM, Litscher SJ, Seitz LL, Messing A, Hagemann TL, Collier LS (2021) Pexidartinib treatment in Alexander disease model mice reduces macrophage numbers and increases glial fibrillary acidic protein levels, yet has minimal impact on other disease phenotypes. J Neuroinflammation 18:67.

Chen JF, Liu K, Hu B, Li RR, Xin W, Chen H, Wang F, Chen L, Li RX, Ren SY, Xiao L, Chan JR, Mei F (2021) Enhancing myelin renewal reverses cognitive dysfunction in a murine model of Alzheimer's disease. Neuron 109:2292-2307.e2295.

Hagemann TL, Powers B, Lin NH, Mohamed AF, Dague KL, Hannah SC, Bachmann G, Mazur C, Rigo F, Olsen AL, Feany MB, Perng MD, Berman RF, Messing A (2021) Antisense therapy in a rat model of Alexander disease reverses GFAP pathology, white matter deficits, and motor impairment. Sci Transl Med 13:eabg4711.

Nasrabady SE, Rizvi B, Goldman JE, Brickman AM (2018) White matter changes in Alzheimer's disease: a focus on myelin and oligodendrocytes. Acta Neuropathol Commun 6:22.

Olabarria M, Putilina M, Riemer EC, Goldman JE (2015) Astrocyte pathology in Alexander disease causes a marked inflammatory environment. Acta Neuropathol 130:469-486.
