# Supplementary figures and images for: Gfap Mutation and Astrocyte Dysfunction Lead to a Neurodegenerative Profile with Impaired Synaptic Plasticity and Cognitive Deficits in a Rat Model of Alexander Disease
Source: eNeuro. 2025 Mar 19;12(3):ENEURO.0504-24.2025. doi: 10.1523/ENEURO.0504-24.2025 (PMC11936449; doi:10.1523/ENEURO.0504-24.2025)

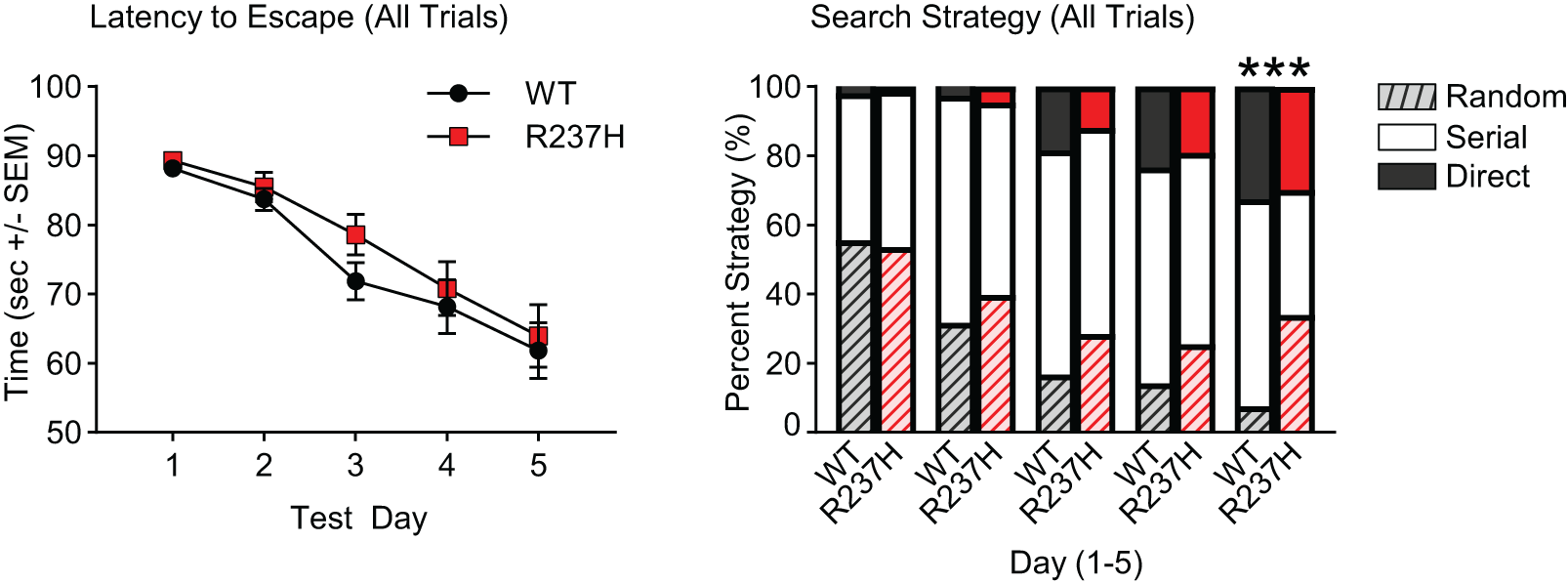

Supplement: Figure 8-1 — Barnes maze performance for wild type and R237H rats. (A) Average latency to escape for 4 trials daily over 5 consecutive days of training, (no significant effect of genotype, mixed-effect models; **p < 0.01, Sidak’s post-test, error bars = SEM). (B) Search strategy patterns (random, serial, direct; see Methods section) used by rats to learn the location of the escape hole in the Barnes maze. Percent strategy used for all trials over each of the 5 training days are shown for comparison between wild type (WT) and R237H rats (Chi Square; ***p<0.001). N = 30 WT, 24 R237H, both sexes at 16 weeks of age for A and B. Download Figure 8-1, TIF file. [file eneuro-12-ENEURO.0504-24.2025-s001.tif]
